# Diabetes Prediction Through Linkage of Causal Discovery and Inference Model with Machine Learning Models

**DOI:** 10.3390/biomedicines13010124

**Published:** 2025-01-07

**Authors:** Mi Jin Noh, Yang Sok Kim

**Affiliations:** 1 Department of Business Big Data, Keimyung University, Daegu 42601, Republic of Korea; mjnoh@kmu.ac.kr; 2 Department of Management Information Systems, Keimyung University, Daegu 42601, Republic of Korea

**Keywords:** diabetes, machine learning, causal discovery, causal inference

## Abstract

**Background/Objectives**: Diabetes is a dangerous disease that is accompanied by various complications, including cardiovascular disease. As the global diabetes population continues to increase, it is crucial to identify its causes. Therefore, we predicted diabetes using an AI model and quantitatively examined causal relationships using a causal discovery and inference model. **Methods**: Kaggle’s dataset from the National Institute of Diabetes and Digestive and Kidney Diseases was analyzed using logistic regression, deep learning, gradient boosting, and decision trees. Causal discovery techniques, such as LiNGAM, were employed to infer relationships between variables. **Results**: The study achieved high accuracy across models using logistic regression (84.84%) and deep learning (84.83%). The causal model highlighted factors such as physical activity, difficulty in walking, and heavy drinking as direct contributors to diabetes. **Conclusions**: By combining AI with causal inference, this study provides both predictive performance and insight into the factors affecting diabetes, paving the way for tailored interventions.

## 1. Introduction

Diabetes mellitus, which is characterized by chronic hyperglycemia, is a serious health problem worldwide. As of 2021, there are 529 million patients with diabetes worldwide, and the global age-standardized diabetes prevalence is 6.1%. Overall, diabetes prevalence mainly reflects type 2 diabetes, accounting for 96 0% of diabetes cases in 2021. It is projected that by 2050, 1.31 billion people will have diabetes [1]. Globally, diabetes imposes a significant health and economic burden. According to a 2019 report from the International Diabetes Federation (IDF), the annual death toll attributed to diabetes exceeded 4 million people. The global annual diabetes-related health expenditure per person is approximately $760 billion, and this is expected to increase to $845 billion by 2045 [2]. These statistics underscore the critical need for effective diabetes management, prevention efforts, and policy development.

Diabetes is a disease that requires consistent management, including medication therapy. As the aging population accelerates, the prevalence of diabetes continues to rise, contributing to a rapid escalation in healthcare costs. Furthermore, due to the rapid aging of the population, diabetes is on an upward trajectory annually, making it challenging to achieve adequate levels of chronic disease management solely through individually focused medical services. Consequently, there is a necessity to systematically manage individualized treatment information for patients with diabetes, enabling the prediction of diabetes risks.

As infectious diseases like COVID-19 continue to spread, the importance of predicting risks for patients with underlying health conditions has grown significantly. These patients are facing challenges not only in their daily lives due to the fear of contagion but are also experiencing heightened physical health. Patients with underlying conditions like diabetes are particularly susceptible to severe stress, depression, and physical fatigue in the wake of infectious disease outbreaks. The experience of intense stress and depression among these patients can exacerbate conditions such as diabetes. Therefore, we would like to study factors that affect the development of diabetes, such as physical and mental factors.

Predicting causality and outcomes in medical fields like diabetes research is a challenging endeavor. Causal inference methodologies provide a means of measuring the extent of effectiveness and can be employed in medical contexts to precisely gauge the efficacy of specific factors. By employing causal inference methodologies, it is possible to assess the effectiveness of interventions for diabetes while considering factors such as psychological influences. This approach could enable the measurement of the impact of health factors on diabetes by considering treatment factors.

Deep learning has contributed to artificial intelligence (AI) systems that accelerate and enhance a wide range of tasks, including decision-making, prediction, anomaly detection, and pattern recognition. The accuracy of deep learning models has improved significantly over the past decade; however, this increased accuracy has also led to greater model complexity. This complexity can render the model unreliable, as it fails to provide a clear explanation for its errors. However, explainable AI (XAI) seeks to address this issue by characterizing the model accuracy, transparency, and the outcomes of AI systems [3]. In essence, XAI methods aim to furnish human-readable explanations that enable users to comprehend and trust the results generated by deep learning algorithms.

Explainable artificial intelligence (XAI) can elucidate the predictions made by machine learning models. Therefore, this study aims to conduct an explainable AI analysis to overcome the limitation that machine learning and deep learning possess complex and nonlinear structures, making them difficult to interpret. This study will utilize machine learning algorithms to predict diabetes and address the limitations of machine learning; it will also perform additional analysis using explainable AI techniques such as causal discovery and causal inference. Causal discovery reveals relationships within raw data, and associative questions can be deduced directly from raw data using traditional statistical techniques. Machine learning shows correlations, while causal inference shows the causal effects of variables within the raw data [4]. Based on these considerations, the central research questions of this study are as follows:

Question 1: Can integrating machine learning models with causal discovery and inference techniques improve the accuracy of diabetes prediction?

Question 2: What are the practical benefits of combining prediction models with causal analysis?

Question 3: Can we measure the quantitative impact of the treatment variable Difficulty Walking on diabetes prediction?

## 2. Materials and Data Description

### 2.1. Artificial Intelligence and Causal Model

Due to the explosive increase in big data and the advancement of hardware technology, research in the fields of artificial intelligence models, such as machine learning and deep learning, is actively underway. The most important aspect of machine learning and deep learning is generalization, but this has been difficult. Current machine learning algorithms learn and evaluate large amounts of data obtained from the same data distribution by dividing them into training data and test data under the assumption that they are independent and identically distributed. One of the important issues is that machine learning algorithms must be generalized so that they can achieve similar performance when learning new data and problems. Most machine learning algorithms ignore or consider important information in the data, such as domain shift and temporal structure, as unnecessary or attempt to solve the generalization problem by learning using large amounts of data, assuming independent and identically distributed data. For this reason, some issues need to be addressed in machine learning [5].

First, it is robustness. Controlling the distribution in real data is difficult. Recent studies demonstrate that combining causal inference methods with machine learning can enhance robustness and generalizability. For example, causal models can effectively address domain shifts and temporal dependencies by controlling changes through intervention. Sahar et al. [6] highlighted that integrating causal inference into machine learning improves performance metrics, even in complex scenarios such as diabetes diagnosis [6]. Additionally, causal inference has shown promise in addressing complex medical challenges by estimating treatment effects and understanding intervention impacts. Recent surveys, such as “Causal Inference in the Medical Domain: A Survey”, highlight the application of these methods in medical domains, providing insights into their use for handling complex medical problems and treatment evaluation [7].

Second, the causality perspective can reveal things about causal relationships that cannot be known from existing machine learning. For instance, in epidemiological studies, machine learning estimators for causal inference, such as Targeted Maximum Likelihood Estimation and Double/Debiased Machine Learning, have been explored to estimate causal effects and analyze treatment impacts effectively. These methods are particularly useful in addressing problems with confounding variables, as discussed in “Machine Learning in Causal Inference for Epidemiology” [8].

Most machine learning models assume data independence, which makes them unsuitable for problems with time-series dependencies, such as stock market forecasting or weather forecasting. For example, a model trained to predict stock prices based solely on recent trends may fail when unexpected events, such as an economic crisis, disrupt the data patterns. To solve these problems, it is necessary to utilize causal inference methods. In existing machine learning, predictions are made based on statistical significance, but in causal models, causal relationships are modeled rather than only looking at high accuracy. This means that by performing intervention, variables that affect the relationship can be discovered, providing deeper insights into causal mechanisms [7,8].

### 2.2. Conceptual Framework

**Figure 1:**
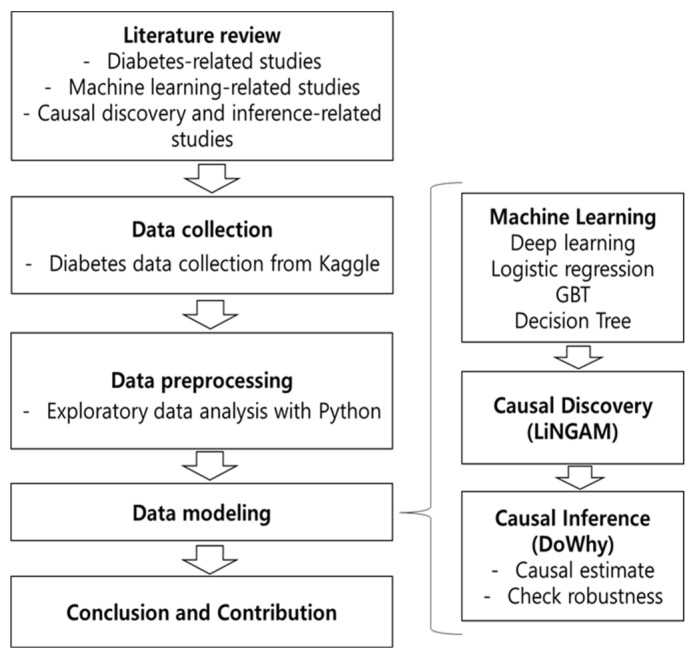
Conceptual framework.

Figure 1 shows the research process. First, it is a literature review. The initial phase involves gathering existing studies and research papers related to machine learning in diabetes and causal inference methods like LiNGAM and DoWhy. The goal of this step is to understand the current situation, identify gaps, and explore relevant research to build on existing knowledge. Second, it is data collection. Following the review, the next step is to collect relevant data sources about diabetes and machine learning applications. This data can include patient records, genetic information, lifestyle factors, and more, which is essential for developing models and conducting causal inference analysis. Third, it is data preprocessing. This phase involves cleaning, organizing, and preparing the collected data for analysis. Tasks like handling missing values, standardizing formats, and ensuring data quality are crucial to ensure the accuracy and reliability of subsequent analyses. Fourth, it is data modeling and analysis like AI models and causal models. Using machine learning techniques, such as predictive modeling or clustering, the preprocessed data is utilized to build models that predict diabetes risk or aid in personalized medicine. These models can uncover patterns and relationships within the data to derive meaningful insights. However, it is difficult to generalize the machine learning model proposed in this study. Therefore, additional analysis is performed based on the causal model. This study uses causal discovery and inference using LiNGAM and DoWhy. Utilizing specialized methods like LiNGAM and DoWhy, the focus shifts to causal inference. These methods help identify causal relationships between variables related to diabetes, offering insights into cause-and-effect relationships within complex datasets. Finally, it is the conclusion and contribution. The culmination of this process involves drawing conclusions based on the findings from both the machine learning analyses and the causal inference methods. Additionally, the limitations of machine learning can be overcome by using intervention variables to identify the cause of changes in diabetes. This final step highlights contributions to the field, potentially unveiling new insights into diabetes management, treatment, or understanding causal relationships in this domain. These contributions could include improved predictive models, identified causal factors, or recommendations for future research directions.

### 2.3. Data Description

We collected the main dataset from Kaggle, and explored and preprocessed the dataset for machine learning modeling and causal modeling. The Supplementary Materials provides data access information. Institute of Diabetes and Digestive and Kidney Diseases, and the patients here are all women aged 21 or older of Pima Indian descent. Table 1 shows information about dataset attributes: BMI, Smoking, Heavy Drinkers, Mental Health, Difficulty Walking, Sex, Age Category, Diabetic, Physical Activity, and General Health.

## 3. Methods

### 3.1. Study Population

Table 2 shows the basic statistical results of this study. The target variable is diabetes data, and the input variables are BMI, Smoking, Heavy Drinkers, Difficulty Walking, Sex, Age Category, Physical Activity, General Health, and Mental Health. The proportion of individuals without diabetes is 84.3% (269,653 individuals), while that of individuals with diabetes is 15.7% (50142 individuals). BMI distribution shows that 67.8% (216,953 individuals) have a low BMI (<30), 19.2% (61,345 individuals) fall into the medium BMI range (30–35), and 13.0% (41,497 individuals) are classified as having a high BMI (>35). The results show that 58.8% (187,887 individuals) identified as non-smokers, while 41.2% (131,908 individuals) are smokers. A total of 93.2% (298,018 individuals) are categorized as heavy drinkers and 6.8% (21,777 individuals) are classified as non-heavy drinkers. A high percentage of the population (86.1%; 275,385 individuals) reported having difficulty walking, whereas 13.9% (44,410 individuals) did not experience walking difficulties. Of these, 52.5% (167,805 individuals) are female, and 47.5% (151,990 individuals) are male. The age distribution shows the largest proportion of individuals in the 60–69 age group (21.2%, 67,837 individuals), followed by the 50–59 age group (17.2%, 55,139 individuals). Physical activity was reported by 77.5% (247,957 individuals) of the population, with 22.5% (71,838 individuals) indicating no engagement in physical activity. Among the general health categories, 35.6% (113,858 individuals) are rated as very good, followed by the good category (29.1%, 93,129 individuals). Low levels of mental health were reported by 75.6% (241,653 individuals), while 24.4% (78,142 individuals) reported high levels of mental health.

### 3.2. Artificial Intelligence Model

Machine learning and deep learning methods have been used to solve several problems, such as cancer, COVID-19, diabetes, and heart disease. Recent research suggests that machine learning shows patient states, predicts risk, and diabetes prediction models [9,10]. In our research, we applied multiple AI algorithms, including deep learning, logistic regression, GBT, and decision trees, to predict diabetes.

Deep learning is a subset of machine learning that allows computers to solve more complex problems. Deep learning models can also create new features themselves. Deep learning can play a pivotal role in healthcare by analyzing trends and behaviors to predict patient illness. In addition, since healthcare workers can use deep learning algorithms to determine the optimal test and treatment for patients, they were used in the analysis to predict diabetes.

Logistic regression is an important technique in the field of artificial intelligence and machine learning (AI/ML). ML models are software programs that can learn to perform complex data-processing tasks without human intervention. In healthcare, this analytical approach can be used to predict the likelihood of a disease in specific populations. Healthcare organizations can establish preventive treatments for individuals with a high incidence of certain diseases. Therefore, logistic regression analysis can be used for diseases such as diabetes.

In machine learning, boosting involves creating a strong model by sequentially applying a weak model multiple times. Gradient boosting is one of the ensemble techniques in machine learning. It is a method of improving the prediction performance of the entire model by repeatedly adding new models that reduce the residuals created by previous models. Gradient boosting has also been used in the medical field to help diagnose various diseases. In particular gradient boosting is particularly useful in medical data analysis due to its ability to capture complex nonlinear relationships and interactions. Gradient boosting is used in breast cancer diagnosis and diabetes prediction.

A decision tree is one of the predictive modeling methods used in statistics, data mining, and machine learning. It uses a decision tree as a prediction model that connects the observed value and target value for an item, and regression tree analysis specifies the predicted result. A real number with meaning can be output. In other words, a decision tree represents choices and results in the form of branches, nodes are attributes of the group to be classified, and branches represent values that the node cannot use. Decision trees can be used to predict diseases such as diabetes.

### 3.3. Model Evaluation

**Figure 2:**
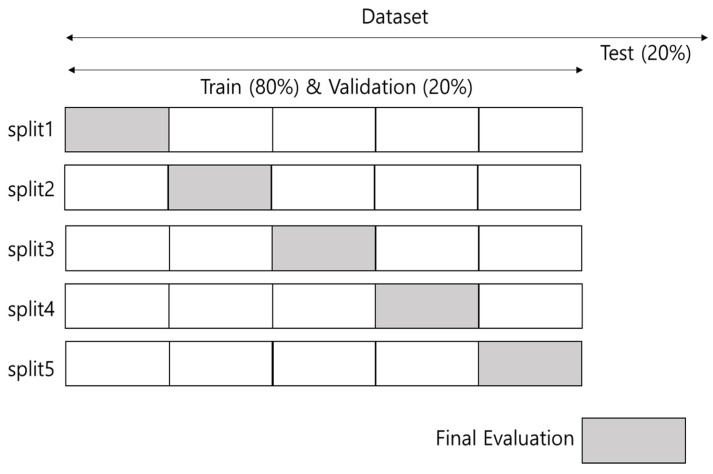
5-fold cross-validation.

K-fold cross-validation is a method for testing the performance of a machine learning predictive model. We use randomized cross-validation (5-fold) to determine the optimum hyperparameters. Figure 2 shows 5-fold cross-validation.

**Figure 3:**
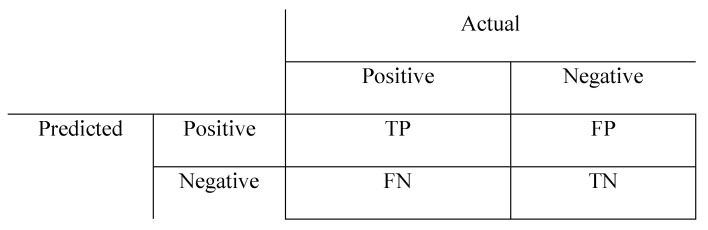
Confusion matrix.

The performance indicators of how well a classification model has been learned include the accuracy, precision, and recall. The performance indicators can be evaluated using a confusion matrix. A confusion matrix is a tool that evaluates a classification model using matching between actual classes and predicted classes. In a binary classification problem, the actual classes are divided into positive and negative, and the classification model classifies the samples into positive and negative. Therefore, four cases occur: TP (True positive), FP (False Positive), FN (False Negative), and TN (True Negative). The confusion matrix is shown in Figure 3. True Positive (TP) refers to cases where the model correctly predicts the positive class. For example, if a patient truly has a disease and the model predicts that they have the disease, this is a true positive. False Positive (FP) occurs when the model incorrectly predicts the positive class for a case that actually belongs to the negative class. For instance, if a patient does not have a disease, but the model predicts that they do, this is a false positive. False Negative (FN) represents cases where the model predicts the negative class for instances that actually belong to the positive class. For example, if a patient has a disease but the model predicts that they do not, this is a false negative. True Negative (TN) describes cases where the model correctly predicts the negative class. For instance, if a patient does not have a disease and the model also predicts no disease, this is a true negative. These components form a confusion matrix, which serves as the foundation for evaluating a classification model.

Accuracy is the ratio of TP to TN among all identified samples. It is the simplest metric for evaluating a classification model.

$$\mathrm{Accuracy}=\frac{TP+TN}{TP+FP+FN+TN}$$

Precision is the ratio of samples that are actually positive to those judged as positive by the classification model.

$$precision=\frac{TP}{TP+FP}$$

Recall is the proportion of actual positive samples judged as positive by the classification model.

$$Recall=\frac{TP}{TP+FN}$$

The area under the curve (AUC) is a measure that combines sensitivity and specificity, while the F1 score balances precision and recall. Additionally, leveraging three separate time windows serves as a mutual model-checking mechanism, ensuring consistency in model performance and preventing overfitting to specific patterns within the window. This approach also improves the robustness of the methodology by systematically evaluating its performance over different follow-up periods.

To enhance the analysis, it is essential to examine the reasons for the variations in the performance indicators between different algorithms. For instance, some algorithms, such as decision trees, may achieve high accuracy, but suffer from overfitting when applied to imbalanced datasets because they focus heavily on the majority class. In contrast, algorithms like logistic regression often achieve a better balance across precision and recall due to their inherent ability to manage class boundaries effectively. Additionally, variations in recall may arise due to differences in algorithmic sensitivity to false negatives. For example, deep learning, when tuned appropriately, may better capture complex patterns in data, resulting in higher recall compared to simpler models like Naive Bayes, which assumes feature independence and may miss such patterns. Error rate differences can also be attributed to how the algorithms handle noise in the data. Ensemble methods such as Gradient Boosting typically have lower error rates because they reduce variance through averaging, whereas single models like k-Nearest Neighbors (k-NN) may be more sensitive to noisy data points.

By analyzing the evolution of these performance metrics, we can understand the strengths and limitations of each algorithm, as well as its suitability for specific applications.

### 3.4. Causal Discovery and Inference

Although artificial intelligence systems are widely used in everyday human activities, the fact that they can only make simple predictions or explain associations is problematic. Therefore, research has been conducted on reliable machine learning tools, and it has been possible to solve some of the machine learning limitations through causal models [11].

Existing causal relationship research has been applied to various fields, such as management, economics, statistics, and computer science, but fragmentary studies that only look at causal relationships have been conducted. However, there are two areas in the causal relationship area: causal discovery and causal inference. Causal discovery attempts to infer causal relationships for various variables in an observational data set. Causal inference focuses on testing whether two variables are related and assessing the impact of one on the other. In other words, causal discovery makes inferences directly from the data set without assuming any relationship between related variables. On the other hand, causal inference assumes a relationship between variables, and attempts to test and quantify the actual relationship using available data. Therefore, to overcome the limitations of machine learning, we additionally analyze causal discovery and causal inference as explanatory methodologies. We try to infer relationships between variables in the data through causal discovery and quantitatively explain the relationships between variables inferred through causal inference.

## 4. Results

### 4.1. Machine Learning

Machine learning (ML) is a methodology that allows system algorithms to learn patterns from data and make predictions or decisions without being explicitly programmed. In this study, machine learning algorithms, including deep learning, logistic regression, GBT, and decision trees, were applied to predict diabetes and evaluate their performance. Table 3 displays the performance metrics for various machine learning algorithms. The accuracy values for deep learning, logistic regression, gradient boosted trees (GBT), and decision trees are approximately 84.83%, 84.84%, 84.66%, and 84.40%, respectively. Looking beyond the accuracy, the other metrics show distinctions among the algorithms. The error rates are relatively similar between logistic regression and deep learning, whereas GBT and decision trees exhibit slightly higher error rates. In terms of the Area Under the Curve (AUC), which evaluates the model’s ability to discriminate between classes, deep learning demonstrates the highest performance at 79.3%, with other algorithms trailing slightly behind. Precision, recall, and F1-score also show nuances between the algorithms. While GBT displays the highest recall value, in other metrics, such as precision and F1-score, deep learning or logistic regression tends to perform better.

Overall, each algorithm excels in different aspects. Deep learning performs well in terms of AUC, logistic regression demonstrates competitive accuracy and precision, GBT shows high recall, and decision trees present a balanced performance across various metrics. The choice of algorithm may depend on the specific priorities or trade-offs concerning these different performance measures.

### 4.2. Causal Discovery Based on LiNGAM (Linear Non-Gaussian Acyclic Model)

LiNGAM is a new method for estimating structural equation models and linear causal Bayesian networks. It is based on using the non-Gaussian nature of the data. Several studies [12] have recently formalized concepts related to causality using probability distributions defined on directed acyclic graphs. This line of research emphasizes the importance of understanding the processes that generate data rather than characterizing the joint distribution of observed variables. The reasoning is that a causal understanding of the data is essential for predicting the outcome of an intervention, such as setting a given variable to a specific value. We utilized a method called LiNGAM to identify linear, non-Gaussian, and non-circular causal models based on data on diabetes.

**Figure 4:**
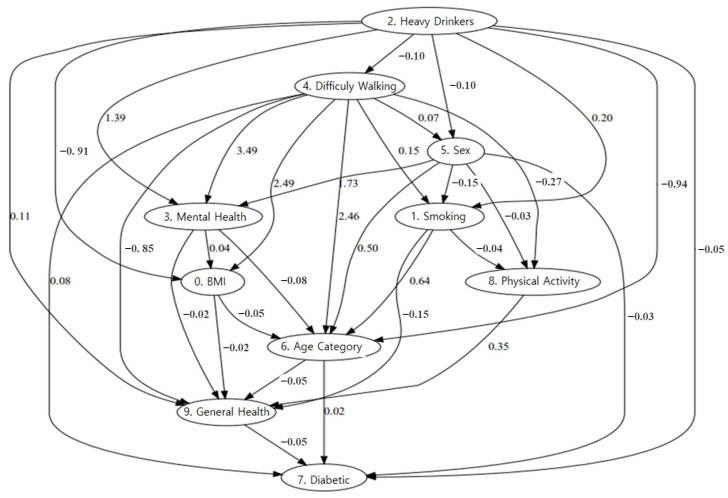
Causal Discovery Graph.

Causal discovery is finding a causal graph based on the preferred direction from observational data, where variables are treated as nodes in the graph and the edges are unknown. We used causal discovery to identify the causal structure of the factors influencing diabetes. In other words, observational data derives causal relationships based on a series of observation data recorded without intervention. Figure 4 shows the results of the causal discovery graph. The relationship between walking difficulty and diabetes in this graph suggests that mobility problems may play an important role in the onset and progression of diabetes. This positive causality indicates that increased walking difficulty may increase the likelihood of diabetes. In addition, walking difficulty is interconnected with other factors such as age, BMI, and overall health. This suggests that the relationship between walking difficulty and diabetes does not exist in isolation but is part of a broader network of influences. Diabetes shows strong associations with key factors, such as age and overall health. Ultimately, walking difficulty appears to contribute to and arise from diabetes and serves as a link between diabetes and other health variables.

### 4.3. Causal Inference

We identified causal relationships between variables using a causal discovery graph, and would like to perform causal inference based on this graph. Causal inference draws a causal graph along edges from intervening variables to the target variables. In a causal model, when treatment is provided, intervention can be used to determine the degree of impact. DoWhy (version 0.12) is software that supports causal inference. DoWhy uses causal graphs to define structured causal relationships and estimate the effectiveness of interventions. DoWhy’s causal inference mechanism consists of four steps. Step 1 draws a causal graph based on the data and visually expresses the causal relationship. Step 2 identifies causal relationships in the model and estimates the causes. Step 3 obtains estimates for the identified causal relationships. Step 4 attempts various refutations of the obtained estimate, which may not be correct. Table 4 shows a summary of the causal inference results.

Step 1 Create a causal graph: We generated a causal graph in the DoWhy software based on the results of causal discovery. We used the DoWhy software to conduct causal inference on diabetes and investigated the influence of mental health as an intervention variable. In the first step, we built a causal model based on causal discovery. In other words, we built a DoWhy-based causal model based on a conceptual network automatically proposed by causal discovery. In the causal model, the input variables are BMI, smoking, heavy drinkers, sex, age category, physical activity, general health, and mental health. The treatment factor is the difficulty walking, and the outcome factor is the diabetes factor.

Step 2 Identify the causal effect: The causal graph properties are used to identify the estimated causal effects. To identify causal effects, mental health was used as an instrumental factor, diabetes was used as an output factor, and the backdoor criterion was used.
identified_estimand = model.identify_effect(proceed_when_unidentifiable = True)
Estimand type: nonparametric-ate ### Estimand: 1 Estimand name: backdoor Estimand expression: d ──────────────(E[Diabetic|AlcoholDrinking]) d[DiffWalking] Estimand assumption 1, Unconfoundedness: If U→{DiffWalking} and U→Diabetic then P(Diabetic|DiffWalking,AlcoholDrinking,U) = P(Diabetic|DiffWalking,AlcoholDrinking) ### Estimand: 2 Estimand name: iv No such variable(s) found! ### Estimand: 3 Estimand name: frontdoor No such variable(s) found!


Step 3 Estimate the identified estimand: DoWhy provides three causal inference methods: backdoor, frontdoor, and instrumental variable approaches. This study utilizes the backdoor criterion, which identifies the causal relationship between treatment variable X and outcome variable Y by conditioning on measured common causes that affect both X and Y. Since this study infers causality from pre-collected observational data without guaranteed pre-treatment equivalence, selection bias may arise. To mitigate such bias, we employ the propensity score method, a statistical approach designed to address this issue. The propensity score represents the probability of assignment to the treatment group versus the control group, given a set of covariates. The three most common methods based on propensity scores are stratification, matching, and weighting. Propensity score stratification involves grouping individuals with similar propensity scores into K strata to ensure comparability between the treatment and control groups within each stratum. Propensity score-matching pairs of individuals from the treatment group with those from the control group with identical or similar propensity scores. Finally, propensity score weighting assigns weights to the treatment and control groups to balance their propensity scores, thereby achieving comparability. Since we need to estimate the effect of the prescription when causality is confirmed, we used propensity score weighting. The estimate_effect function was used, and the effect of the prescription was estimated using ATE (Average Treatment Effect). As a result, the incidence of diabetes among patients who had difficulty walking was found to increase by about 22.5%.
estimate = model.estimate_effect(identified_estimand, method_name = “backdoor.propensity_score_weighting”, target_units = “ate”)
## Estimate Mean value: 0.22503235811900396


### 4.4. Refutation

To check whether the assumption using causal relationships is accurate, we perform a refutation test on the results in three ways. Refutation testing is a method of testing how the results change by changing the model or data structure. DoWhy provides Random common cause, Placebo Treatment Refuter, and Data Subset Refuter methods. The Random Common Cause method adds randomly drawn covariates to the data and reruns the analysis to see whether the causal estimates change. If our assumptions were originally correct, our causal estimates would not change significantly. The results of this study appear to be satisfactory, as there is little change in the causal estimates of the estimated effect and new effect. The Placebo Treatment Refuter method randomly assigns any covariate to the treatment and reruns the analysis. Looking at the results, the *p*-value is 0.94; therefore, there appears to be no problem with the causal estimate. The Data Subset Refuter method creates subsets of data (similar to cross-validation) and determines whether causal estimates differ across subsets. The results showed that there was no problem with the causal estimate. Since the refutation test showed that there were no problems with the estimate, it can be said that the reliability of the estimate was secured. Table 5 shows a summary of the refuted results.

## 5. Discussion

In this study, machine learning modeling was performed to identify the importance of diabetes diagnosis and effective management. Machine learning modeling showed excellent performance indicators. For example, the logistic regression model (84.84%) and deep learning model (84.83%) showed high accuracy, and the logistic regression model (85.94%) also showed high precision. GBT (99.19%) and decision tree (98.82%) showed high recall. Most machine learning models for diabetes prediction have shown high-performance indicators. Previous studies have emphasized the importance of diabetes prediction and proposed machine learning diabetes prediction models [13]. In this study, diabetes prediction was performed using machine learning algorithms emphasized in previous studies, and excellent prediction models such as deep learning, logistic regression model, GBT, and decision tree were proposed.

However, the limitations of traditional machine learning prediction models have been suggested, and to solve this, the possibility of diabetes prediction was proposed by integrating machine learning algorithms and causal inference algorithms. While machine learning effectively predicts outcomes based on data patterns, it often falls short of identifying causal relationships that are essential for understanding disease mechanisms and guiding effective interventions. Previous studies have highlighted the effectiveness of integrating causal inference and machine learning to improve diabetes diagnosis and management. However, the research results focus on enhancing machine learning performance indicators [6].

In contrast, this study demonstrated the effectiveness of improving machine learning performance and causal inference. The strength of the approach proposed in this study is that it can discover direct and indirect causal paths for diabetes prediction. By utilizing causal inference methodologies, such as LiNGAM and DoWhy, it was shown that health-related parameters, such as BMI and age, and physical activity, such as walking, are both causes and consequences of diabetes. This dual relationship emphasizes the importance of the intervention of treatment variables that can alleviate diabetes risk and progression. Causal inference also demonstrated that interventions to improve physical activity and mobility can reduce the likelihood of diabetes, as evidenced by an average treatment effect (ATE) estimate of 0.23. By applying causal discovery and inference techniques along with machine learning, this study successfully identified causal diagrams that contribute to diabetes development. For instance, while machine learning accurately predicted diabetes risk, causal inference revealed that specific interventions, such as increasing physical activity and improving mobility, effectively reduced this risk. This combined approach bridges the gap between predictive modeling and actionable insights, where traditional machine learning often struggles to explain intervention effects.

A significant contribution of this study is its emphasis on environmental and lifestyle factors. Previous studies on type 2 diabetes have demonstrated that physical activity and walking exercise are important factors in reducing the risk of diabetes [14]. Our results also comprehensively demonstrated the positive influence of physical activity, such as exercise habits, physical activity, and walking ability, on reducing the risk of diabetes. Unlike previous studies that relied only on clinical data, our holistic approach to lifestyle emphasizes the importance of environmental factors in diabetes prevention. Furthermore, causal discovery effectively addressed confounding variables, which often obscure true causal relationships in healthcare data. This study uncovered robust data-driven relationships by employing causal algorithms, mitigating the limitations posed by confounders in traditional analyses.

The proposed methodology also demonstrates broader applicability. Rather than using traditional machine learning algorithms to predict various diseases such as obesity, hypertension, and diabetes, the integrated model proposed in this study shows superiority in predicting various diseases such as cardiovascular disease, hypertension, and obesity. This framework will enable healthcare professionals and related researchers to identify causal relationships and actionable interventions, which can be applied not only in research related to diseases but also in practice. In addition, by utilizing predictive modeling and causal inference to improve prediction accuracy and obtain broader insights into diseases, the causes of diseases can be identified and utilized for disease improvement. Future studies could use real-world data to enhance the robustness and reliability of the integrated model. Finally, longitudinal studies can clearly define intervention factors for the integrated model and identify factors that can prevent diseases.

## 6. Conclusions

This study combined machine learning, deep learning, and causal algorithms to predict diabetes and identify its causal factors, addressing the key limitations of existing predictive models. First, the study enhanced prediction accuracy while uncovering causal relationships. By integrating causal discovery and inference techniques with machine learning, these findings provide deeper insights into the causal mechanisms underlying diabetes. Second, this study highlights the significant role of lifestyle and environmental factors in the prevention of diabetes. Variables such as physical activity and walking ability have been shown to have a substantial impact on diabetes risk. Third, the proposed methodology demonstrates broader applicability across medical research. The integration of machine learning and causal inference can be extended to other chronic diseases, such as cardiovascular disease, hypertension, and obesity. Despite its contributions, this study has some limitations. The data were limited to patients with diabetesfrom 2022. Future research should incorporate more recent and diverse datasets to validate and refine these findings. Additionally, applying this framework to other diseases will further demonstrate its generalizability and contribute to comprehensive data-driven healthcare solutions.

## Figures and Tables

**Table 1:** Variables description of the dataset.

Variables	Description
BMI	Body Mass Index
Smoking	Have you smoked at least 100 cigarettes in your entire life? (The answer Yes or No)
Heavy drinkers	Heavy drinkers (adult men having more than 14 drinks per week and adult women having more than seven drinks per week)
Mental Health	Thinking about your mental health, for how many days during the past 30 days was your mental health not good? (0–30 days)
Difficulty Walking	Do you have serious difficulty walking or climbing stairs?
Sex	Are you male or female?
Age Category	Fourteen-level age category
Diabetic	(Ever told) (you had) diabetes?
Physical Activity	Adults who reported doing physical activity or exercise during the past 30 days other than their regular job
General Health	Would you say that, in general, your health is…

**Table 2:** Characteristics of the study participants.

Variables			Frequency	Percentage (%)
Target variable	Diabetes	no diabetes	269,653	84.3
		diabetes	50,142	15.7
Input variables	BMI	Low (BMI < 30)	216,953	67.8
		Medium (30 < BMI < 35)	61,345	19.2
		High (BMI > 35)	41,497	13.0
	Smoking	Yes	131,908	41.2
		No	187,887	58.8
	Heavy Drinkers	Yes	298,018	93.2
		No	21,777	6.8
	Difficulty Walking	Yes	275,385	86.1
		No	44,410	13.9
	Sex	Male	151,990	47.5
		Female	167,805	52.5
	Age Category	18–29	38,019	11.9
		30–39	39,303	12.3
		40–49	42,797	13.4
		50–59	55,139	17.2
		60–69	67,837	21.2
		70–79	52,547	16.4
		80 or older	24,153	7.6
	Physical Activity	Yes	247,957	77.5
		No	71,838	22.5
	General Health	Excellent	66,842	20.9
		Very good	113,858	35.6
		Good	93,129	29.1
		Fair	34,677	10.8
		Poor	11,289	3.5
	Mental Health	Low	241,653	75.6
		High	78,142	24.4

**Table 3:** Performance of Machine Learning.

	Deep Learning	Logistic Regression	GBT	Decision Tree
accuracy	84.83%	84.84%	84.66%	84.40%
precision	85.63%	85.94%	85.09%	85.09%
recall	98.55%	98.07%	99.19%	98.82%
error	15.17%	15.16%	15.34%	15.60%
AUC	79.30%	79.10%	78.40%	73.80%
F1	91.64%	91.61%	91.60%	91.44%

**Table 4:** Summary of causal inference results.

Research Process	Description
Input data	319,795 entries with 11 columns
Components based on causal graph	Input factors: BMI, Smoking, Heavy Drinkers, Sex, Age Category, Physical Activity, General Health, and Mental Health
	Treatment factor: Difficulty Walking
	Output factor: Diabetes
Identify the causal effect	Estimand type: Backdoor criterion
Estimate the identified estimand	Method: backdoor.propensity_score_weighting
	Target units: ATE
	Estimate: about 0.23
Refute	Add random common cause: *p*-value 0.88
	Placebo treatment: *p*-value 0.94
	Subset of data: *p*-value 0.98

**Table 5:** Summary of Refute results.

Method	Estimated Effect	New Effect	*p*-Value
Random Common Cause	0.2250	0.2250	0.8800
Placebo Treatment Refuter	0.2250	0.0008	0.9400
Date Subset Refuter	0.2250	0.2250	0.9800

## Data Availability

Data are available in a publicly accessible repository.

## Supplementary Materials

The following supporting information can be downloaded at: https://www.kaggle.com/datasets/kamilpytlak/personal-key-indicators-of-heart-disease, Diabetes Dataset. accessed on 1 July 2024.
